# Thiol-Functionalized TiO_2_ as Reactive Nanoadsorbents for Residual Monomer Removal from Waterborne Polymer Dispersions

**DOI:** 10.1021/acs.iecr.5c04301

**Published:** 2026-01-19

**Authors:** Ana Trajcheva, Pablo Morales, Justine Elgoyhen, Radmila Tomovska

**Affiliations:** † POLYMAT and Departamento de Química Aplicada, Facultad de Ciencias Químicas, University of the Basque Country UPV/EHU, Joxe Mari Korta Zentroa, Tolosa Etorbidea 72, Donostia, San Sebastián 20018, Spain; ‡ IKERBASQUE, Basque Foundation for Science, Plaza Euskadi 5, 48009 Bilbao, Spain

## Abstract

This study explores the use of thiol-functionalized TiO_2_ nanoparticles to reduce residual methyl methacrylate (MMA) and butyl acrylate (BA) monomers in waterborne polymer dispersions (latexes). TiO_2_ nanoparticles were modified with (3-mercaptopropyl)­triethoxysilane (MPTES) to introduce −SH groups capable of thiol–ene type reaction with C=C bonds of monomers. The effects of functionalization density, nanoparticle concentration, and mixing time on monomer removal were systematically evaluated. Compared with unmodified TiO_2_, MPTES-modified nanoparticles achieved up to 90% removal of MMA and nearly complete removal of BA, lowering total residual monomer content from ∼1900 ppm to 120 ppm. About 80–90% of the adsorbed monomers were chemically, thus, irreversibly bonded to the TiO_2_ surface. DLS, TEM, and zeta potential analyses revealed improved dispersion and reduced aggregation of modified nanoparticles in the latex, leading to uniform distribution in films obtained by water evaporation. TEM imaging further showed preferential localization of modified TiO_2_ at the polymer particle surfaces, enabling efficient monomer capture while maintaining good dispersion. The resulting films displayed unchanged water sensitivity but significantly improved mechanical properties, with Young’s modulus increasing by 75%. Overall, thiol-functionalized TiO_2_ nanoparticles act as reactive, multifunctional adsorbents that effectively reduce residual monomer content and enhance the mechanical performance of MMA/BA polymer latex coatings.

## Graphical abstract

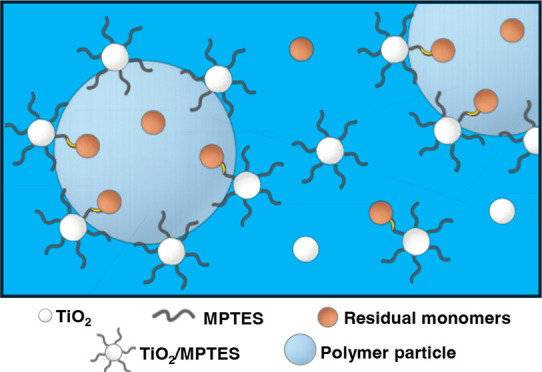

## Introduction

Waterborne polymer dispersions (latexes) are primary products of emulsion polymerization and are gaining increasing industrial interest due to their environmentally benign character and broad application potential. However, a persistent challenge with these materials is the presence of residual monomers, which can remain at levels of up to several percent and are typically released into the atmosphere during latex application. Reducing these residual monomer levels is critical, as they contribute directly to the overall volatile organic compound (VOC) content of the final product. Growing environmental and health concerns, coupled with increasingly stringent regulatory standards,^1^ have therefore made the minimization of residual monomer content a key objective in the development of more sustainable, low-emission coating technologies.

Several strategies have been developed to reduce residual monomer content in latex dispersions. Among the most widely used are postpolymerization treatments based on redox initiator systems (e.g., persulfate/ascorbic acid) introduced after the main polymerization step.^2–5^ While effective in promoting additional monomer conversion, these approaches often lead to the formation of secondary VOCs, such as acetone, and other low molecular weight byproducts, complicating both formulation compliance and environmental safety.^2,5^ Consequently, an additional purification step, most commonly devolatilization,^4,6^ is typically required. This technique employs high temperatures, vacuum conditions, or steam stripping to eliminate both unreacted monomers and newly generated low-molecular-weight VOCs. Although effective, devolatilization poses significant challenges: it is energy-intensive, expensive, and technically complex, especially when applied to aqueous polymer systems. Moreover, the water extracted during the process contains VOCs, requiring further treatment to meet environmental compliance standards.

As an alternative, adsorption^7–10^ has also been investigated. In this approach, latex is passed through packed beds containing adsorbents such as zeolites,^9^ activated carbon,^10^ or ion-exchange resins.^7,8^ These materials capture VOCs through surface interactions, providing high efficiency at low concentrations. Nonetheless, adsorption has practical drawbacks: it requires dedicated purification equipment, is strongly influenced by humidity and temperature, and produces contaminated adsorbents that must be regenerated or disposed of, limiting its scalability and cost-effectiveness.

The adsorption of residual monomers from polymer latexes could have significant practical value if a component already present in a complex coating formulation could simultaneously serve as an adsorbent. In this context, TiO_2_ is particularly noteworthy, as it is extensively used as a white pigment in waterborne coatings. Owing to its high refractive index and exceptional brightness, TiO_2_ provides excellent opacity, color retention, and durability, making it indispensable in both architectural and industrial formulations. These unique optical properties have firmly established TiO_2_ as a critical ingredient in modern coating technologies. Given its widespread use in pigmented coatings and its demonstrated ability to adsorb various hazardous compounds, this work introduces a novel concept: employing TiO_2_ as an in situ adsorbent for residual monomers in polymer latexes intended for waterborne coatings. TiO_2_ offers a high surface area, excellent chemical stability, and proven capacity to adsorb a wide range of toxic organic^11–21^ and inorganic^22–28^ compounds, making it an effective adsorbent in both environmental and industrial applications. Although TiO_2_ is well recognized for its adsorption capabilities in environmental treatments, its direct use for residual monomer removal within polymer latexes has not been previously investigated.

A key limitation of this approach, however, is the potential re-emission of adsorbed monomers during film formation from the polymer latex. Photocatalytic degradation under UV irradiation could, in principle, mitigate this issue, but UV penetration in polymer dispersions is severely limited, as demonstrated in our previous work.^29^

To achieve stable attachment of residual monomers onto TiO_2_, adsorption was combined with a subsequent chemical reaction. Our recent work showed that thiol groups can spontaneously generate thiyl radicals in aqueous media through oxidation, enabling their addition to C=C double bonds of vinyl monomers.^30^ Based on this, we hypothesized that introducing thiol functionalities onto TiO_2_ could enable direct chemical capture of monomers from polymer latexes. 3-(Mercaptopropyl)­triethoxysilane (MPTES) was selected as the coupling agent because it not only provides robust silane anchoring to TiO_2_, as demonstrated previously,^31^ but also introduces surface −SH groups. These thiols promote thiyl radical formation,^32^ allowing TiO_2_ to actively react with monomers rather than serving solely as a physical adsorbent, suppressing their volatilization during film formation. Importantly, TiO_2_ nanoparticles bearing chemically anchored MMA and BA molecules will remain within the aqueous dispersion and later in the dried coating film, where their presence may be beneficial while still functioning conventionally as a white pigment.

This work therefore proposes MPTES-functionalized TiO_2_ as an effective strategy for monomer removal in pigmented waterborne coatings. We hypothesize that surface thiols generate thiyl radicals capable of covalently immobilizing monomers, providing irreversible chemisorption rather than reversible adsorption, and that removal efficiency increases with thiol surface density. Moreover, the process operates at room temperature, requires no external redox initiators, and avoids secondary VOC formation, offering a practical and environmentally friendly approach to emission reduction in waterborne coatings.

## Experimental Section

### Materials

Methyl methacrylate (MMA) and butyl acrylate (BA) of technical grade were obtained from Quimidroga and utilized directly, without any additional purification steps. Emulsion polymerization was stabilized colloidally using alkyldiphenyloxide disulfonate (Dowfax 2A1, 45% active ingredient), generously provided by Dow Chemicals, as the surfactant. Potassium persulfate (KPS, Sigma-Aldrich) was employed as the thermal initiator, while deionized water acted as the continuous medium throughout the polymerization reaction.

Anatase-phase titanium dioxide nanoparticles (TiO_2_, ∼20 nm, surface area 45–55 m^2^/g) were purchased from Sigma-Aldrich and used as received. Surface modification of TiO_2_ was performed using 3-mercaptopropyltriethoxysilane (MPTES, 97%, abcr GmbH), with 2-butanone (Sigma-Aldrich) serving as the reaction solvent.

For molecular weight determination by size exclusion chromatography (SEC/GPC), GPC-grade tetrahydrofuran (THF, 99.9%, Scharlab) was employed to ensure high analytical accuracy. Dimethyl sulfoxide (DMSO, 99.9%, Thermo Scientific) was used as the solvent for gas chromatography (GC) analysis. 1-Pentanol (99%, Sigma-Aldrich) was used as an external standard in GC measurements.

### Preparation of MMA and BA Aqueous Monomer Solution

An aqueous solution containing MMA and BA monomers was prepared considering their solubility limits, 1.5 g/L for MMA and 0.3 g/L for BA. Approximately 0.2 g of each monomer was dissolved in 500 mL of Milli-Q water. The mixture was stirred at 250 rpm for 30 min, yielding final monomer concentrations of 525 ppm for MMA and 502 ppm for BA. For clarity, the term “aqueous monomer solution” will hereafter refer to water with dissolved MMA and BA monomers.

### Synthesis of MMA/BA Polymer Latex

Polymerization of a 50:50 weight ratio of MMA and BA was carried out through a semicontinuous, two-stage seeded emulsion polymerization method. Initially, a seed with 20% solids was synthesized by batch polymerization in a 2 L jacketed glass reactor. The reactor setup included a reflux condenser, nitrogen inlet, several feed lines, and a stainless-steel anchor stirrer with six blades operating at 220 rpm. The reactor charge consisted of deionized water, the MMA/BA monomers, and Dowfax 21A surfactant. The mixture was heated to 80 °C before rapid addition of an aqueous KPS initiator solution to commence polymerization, which was maintained for 2 h to generate the seed.

Subsequently, the polymerization was continued to reach approximately 50% solids in the final latex. Additional KPS initiator was added to the seed batch, and a pre-emulsified mixture of MMA, BA, surfactant, and water was fed gradually over 3 h via an automated dosing pump controller via Camile TG, CRW Automation Solutions. After completing the monomer feed, the reaction temperature was held at 80 °C for one more hour to achieve full conversion of monomers. The specific formulations for both stages are detailed in Table S1 (Supporting Information).

### Surface Modification of TiO_2_ with MPTES

TiO_2_ nanoparticles were functionalized with MPTES (Scheme S1, Supporting Information) based on a previously established protocol developed in our earlier work.^31^ Based on the methodology previous study, two MPTES/TiO_2_ ratios (1 g/g and 6 g/g) were selected, as they had previously shown the most effective surface modification, producing the largest increase in hydrophobicity and indicating optimal silane grafting efficiency. The modification was carried out in 2-butanone, which served as the dispersion medium. The MPTES/TiO_2_ mixtures were sonicated for 40 min at 70% amplitude, using a pulsed cycle of 0.5 s ON/0.5 s OFF, without external cooling. Full experimental compositions and conditions are detailed in Table S2 (Supporting Information).

To remove the free MPTES, the dispersions were subjected to a purification process consisting of centrifugation at 15,000 rpm for 15 min, followed by washing the sediment with acetone. This wash–centrifugation cycle was repeated three times to ensure thorough removal of free MPTES. The resulting modified TiO_2_ was then dried at 65 °C for 24 h in a convection oven prior to further use.

### Preparation of 20 wt % MPTES/TiO_2_ Water Dispersions

Three distinct 20 wt % aqueous dispersions were prepared: one containing unmodified TiO_2_ (0 g/g MPTES/TiO_2_) and two containing modified TiO_2_ at 1 g/g and 6 g/g MPTES/TiO_2_ ratios (Table S3). The dispersions were homogenized by ultrasonication for 30 min (1 s ON/1 s OFF cycle, 75% amplitude) in an ice bath. The pH was adjusted to 11 using 1 M NaOH to ensure optimal dispersion stability.

### Preparation of Blends TiO_2_/Monomer Solution or TiO_2_/Polymer Latex

Blending was carried out by gradually adding 20 wt % aqueous dispersions of either MPTES-modified or unmodified TiO_2_ to aqueous monomer solution or to the MMA/BA latex, under continuous stirring at room temperature. A dropwise addition method was used to ensure uniform mixing. The volume of dispersion added was adjusted to achieve final TiO_2_ concentrations of 0.2, 1, and 2 wt %, calculated relative to the total mass of the latex or aqueous monomer solution (see Tables S4 and S5).

To investigate the influence of contact time on the adsorption of residual monomers onto the TiO_2_ surface, blending was carried out over three different durations: 1, 3, and 5 h. This allowed for evaluation of the time-dependent interaction between the TiO_2_ and the monomer species remaining in the latex.

### Characterization

#### Particle Size and Distribution Analysis

The mean particle size and distribution of the latex samples was measured by dynamic light scattering (DLS) using a Zetasizer Nano Z instrument (Malvern Instruments). For each analysis, one drop of latex or TiO_2_ dispersions was dispersed in 4 mL of deionized water to prepare the sample.

#### Monomer Conversion

Monomer conversion was assessed gravimetrically. A small volume of latex was distributed into five aluminum cups, each prefilled with a 0.1% hydroquinone solution to inhibit any further polymerization. The samples were then placed in an oven and dried at 60 °C for 24 h. Following drying, the cups were weighed again to determine the solid content (SC) of the latex, calculated according to eq 1. This SC value was then used to estimate the monomer conversion, as described in eq 2, which relates the total formulation mass (tot) to the mass of monomers (*M*).

1 $$SC\;\left(\%\right)=\frac{dry\;polymer}{latex}\times 100$$

2 $$conversion\;\left(\%\right)=\frac{SC\times tot}{M}\times 100$$

#### Zeta Potential Measurements

The zeta potential of the MMA/BA latex, as well as of both unmodified and MPTES-modified TiO_2_ nanoparticles, was measured using a Zetasizer Nano ZS (Malvern Panalytical). This technique calculates zeta potential based on particle electrophoretic mobility, obtained through laser Doppler velocimetry combined with phase analysis light scattering (M3-PALS). Separate dispersions of 0.05 wt % polymer latex and 0.05 wt % TiO_2_ (modified and unmodified) were prepared in deionized water at a basic pH of approximately 11, adjusted by adding NaOH. To study the effect of pH on surface charge, the pH of these dispersions was gradually lowered by adding 0.05 M HCl, and corresponding zeta potential values were recorded. All measurements were conducted at a controlled temperature of 25 °C, with samples equilibrated for 60 s prior to data acquisition. Samples were loaded into capillary cells equipped with electrodes, across which an electric potential was applied to induce particle movement for mobility analysis.

#### Fourier-Transform Infrared Spectroscopy (FTIR)

FTIR was employed to verify the success of the TiO_2_ surface modification with MPTES. Spectra were recorded using a Bruker Alpha FTIR spectrometer equipped with an attenuated total reflection (ATR) accessory. A small quantity of both functionalized and unmodified TiO_2_ powders was placed directly onto the ATR crystal. The measurements were conducted in absorption mode over the spectral range of 3000–400 cm^–1^, with a spectral resolution of 0.9 cm^–1^. Characteristic vibrational bands associated with silane groups were monitored to confirm successful surface functionalization.

#### Contact Angle Measurements

Static water contact angle measurements were performed using a Contact Angle System OCA (Data physics Instruments) to evaluate the surface wettability of the modified and unmodified TiO_2_. The measurements were conducted on compressed TiO_2_ pellets, which were fabricated using an Atlas Manual Hydraulic Pellet Press to ensure a uniform and flat surface. A 7 μL droplet of deionized water was gently deposited onto the pellet surface, and the contact angle was recorded 15 s after deposition to allow for stabilization. Each sample was measured at five different locations to ensure reproducibility and account for surface variability.

#### Residual Monomer Quantification by Gas Chromatography

Residual monomers were quantified using direct injection gas chromatography (DIGC) on an HP 6890 system equipped with a flame ionization detector (FID). The chromatographic conditions were as follows: injector temperature at 230 °C, detector at 280 °C, helium as the carrier gas flowing at 5 mL/min, and injector pressure set to 3.8 psi. Separation was achieved using a polar capillary column (SGE BP-21, 30 m × 0.53 mm ID). The oven temperature program started with a 6 min hold at 50 °C, followed by a ramp to 80 °C at 10 °C/min (held for 3 min), then increased to 240 °C at 40 °C/min, and held for 5 min. Data acquisition and processing were performed using ChemStation software.

Calibration curves for MMA and BA were constructed over a concentration range of 1 to 7000 ppm. A stock solution containing 7000 ppm of each monomer was prepared in DMSO and serially diluted to generate the calibration standards. Each 10 g calibration solution was dosed with 2 mg/g 1-pentanol (external standard) and 5 mg/g hydroquinone (0.1 wt % aqueous dispersion) to inhibit radical polymerization. After 30 min of stirring, 5 μL of each standard was injected into the GC. Calibration was performed in duplicate at each concentration level, with relative standard deviation (RSD) values below 5% for both MMA and BA, confirming good reproducibility (see Figure S1).

For sample preparation, latex dispersions were first diluted to 5 wt % with deionized water and centrifuged at 5000 rpm for 30 min to remove TiO_2_ particles, which could otherwise interfere with GC analysis. The resulting supernatants were filtered through a 0.45 mm membrane and further diluted to 2 wt % with DMSO to facilitate monomer partitioning from the polymer matrix. Finally, 1-pentanol (2 mg/g) and hydroquinone (5 mg/g) were added to the samples, which were stirred for 30 min prior to injecting 5 μL aliquots for chromatographic analysis.

#### Desorption of Monomers from Loaded TiO_2_

To differentiate between physically adsorbed and chemically bound monomers on TiO_2_, a solvent extraction method was employed. TiO_2_ nanoparticles previously exposed to monomers were first separated from their respective systems—either aqueous monomer solution or MMA/BA latex—via centrifugation (34,000 rpm for the aqueous solution and 7000 rpm for the latex, both for 10 min). To recover physically adsorbed monomers, solvents were introduced to the monomer-loaded TiO_2_. The goal was to extract only physically adsorbed monomers, while chemically bonded would remain anchored to the TiO_2_ surface. Three solvents—acetone, THF, and ethanol—were tested for this purpose. Acetone and THF were found to be unsuitable due to their poor ability to redisperse TiO_2_, which led to visible coagulation. In contrast, ethanol proved effective in redistributing the TiO_2_ particles without inducing aggregation. After ethanol was added, the dispersion was subjected to ultrasonication to enhance redispersion and facilitate desorption of physically adsorbed monomers. The mixture was then centrifuged, and the ethanol supernatant was analyzed by GC to quantify the desorbed monomer content. This procedure allowed estimation of the physically adsorbed monomer fraction, while those not desorbed were considered to be chemically bonded to the modified TiO_2_ surface.

#### Transmission Electron Microscopy (TEM)

TEM was used for the analysis of the blends for a better understanding of the interaction between the polymer particles and TiO_2_ nanoparticles. TEM analysis was performed using a TECNAI G2 20 TWIN transmission electron microscope operated at 200 kV and equipped with a LaB_6_ filament for enhanced image resolution. Samples were prepared by dispersing the latex or nanoparticle suspension in deionized water. A drop of the diluted suspension was deposited onto a 300-mesh copper TEM grid coated with a pure carbon film. Prior to sample deposition, the grid was glow-discharged to improve wettability and ensure uniform spreading of the sample. The grids were then dried at ambient temperature before imaging.

#### Scanning Electron Microscopy (SEM) Combined with Energy Dispersive X-ray (EDX) Mapping was Employed to Investigate the Distribution of TiO_2_ Nanoparticles within the Polymer Matrix

Polymer films containing either unmodified or MPTES-modified TiO_2_ were analyzed to assess the dispersion and localization of the inorganic phase. All samples were mounted on aluminum stubs using carbon double-sided adhesive tape. Prior to imaging, the specimens were coated with a thin layer of gold (20–25 nm) using an EMITECH K550x metalliser operating at a current intensity of 25 A, in order to improve surface conductivity and image resolution. SEM analysis was conducted using a JEOL JSM-6400 scanning electron microscope equipped with an INCA X-sight Series Si­(Li) pentaFET EDX microanalysis system (Oxford Instruments). Measurements were performed in high vacuum mode at an accelerating voltage of 20 kV and a beam current of 1 nA, with a working distance maintained at 15 mm.

#### Mechanical Properties

The tensile properties of the films, both in the absence and presence of TiO_2_, were assessed using an A.HD Plus texture analyzer (Stable Micro Systems Ltd., Godalming, UK). Dried films were shaped into standardized “dog-bone” specimens (15 mm × 3.5 mm × 0.5 mm) for testing. Uniaxial tensile measurements were carried out at a constant crosshead speed of 1.5 mm/s, equivalent to a nominal strain rate of 0.1 Hz.

#### Water Uptake

To evaluate water uptake, the dry mass of each film sample (*m*_0_) was recorded prior to immersion in distilled water. The dry films had dimensions of 15 mm × 3.5 mm × 0.5 mm. Samples were immersed in distilled water maintained at room temperature (23–25 °C). At predetermined time intervals, the films were removed, gently blotted to remove surface moisture, weighed (m_t_), and then returned to the water. The percentage of water uptake was calculated using the following equation

3 $$Water\;uptake\;\left(\%\right)=\frac{m_{t}-m_{0}}{m_{0}}\times 100$$

All measurements represent the average of three independent replicates to ensure reproducibility.

## Results and Discussion

Initially, the physical adsorption of residual monomers by unmodified TiO_2_ nanoparticles was investigated using an aqueous monomer solution containing 525 ppm of MMA and 502 ppm of BA. TiO_2_ nanoparticles were added to the aqueous MMA/BA solution and stirred for 5 h to allow monomer adsorption. The monomer-loaded TiO_2_ particles were then separated by centrifugation at 5000 rpm for 30 min. Approximately 5% of the initial monomer solution (1.6 mL out of 20 mL) remained associated with the TiO_2_ particles, as complete removal would have required heating, which might have caused premature monomer evaporation.

A procedure was subsequently developed to assess whether the adsorbed monomers could readily desorb and evaporate from the TiO_2_ particles, as described in the Supporting Information. Briefly, two sealed vials were prepared, a control vial containing 1.6 mL of saturated MMA/BA solution, and a vial containing the TiO_2_ nanoparticles with the retained 1.6 mL of aqueous monomer solution after adsorption (Figure S2). Both vials were stored at 25 °C for 4 days. After this period, the vapor phase in the headspace of each vial was quantitatively analyzed by GC. As shown in Figure S2, the chromatographic profile of the vapors from the vial containing loaded TiO_2_ exhibited a similar presence of MMA and distinctly higher presence of BA in the vapor phase (blue curve) compared to the control vial containing only the aqueous solution (black curve). The greater abundance of monomers, particularly BA, indicates that the monomers initially adsorbed onto the TiO_2_ nanoparticles were gradually released over time.

### Surface Modification of TiO_2_ Nanoparticles

To address this limitation, the potential for chemisorption of the monomers through stable covalent attachment to the TiO_2_ surface was investigated. Thiol functionalities are known to spontaneously generate thiyl radicals via atmospheric oxidation, which can initiate thiol–ene type reaction with the C=C double bonds of the monomers.^30^ Based on this principle, TiO_2_ nanoparticles were surface-functionalized with the silane coupling agent MPTES, which contains a −SH moiety (Scheme S1).

TiO_2_ nanoparticles were modified with MPTES by ultrasound induced reaction, at two different MPTES/TiO_2_ mass ratios, 1 and 6 g/g, following a methodology developed previously.^31^ Accordingly, ethoxy groups (−OCH_2_CH_3_) of MPTES undergo hydrolysis and subsequently react with the surface −OH groups abundantly present on native TiO_2_, forming siloxane bonds while leaving the propyl chain terminated with a thiol (−SH) group exposed. The surface modification was evaluated by FTIR, with the resulting spectra presented in Figure 1 and chemical moiety attributed to the observed characteristic absorption bands are summarized in Table S6.

**Figure 1:**
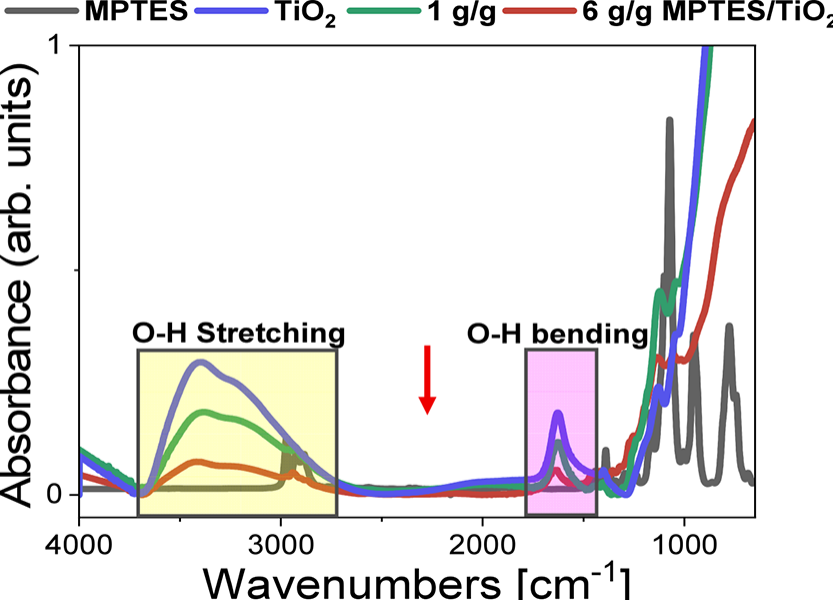
FTIR spectra of TiO_2_, MPTES and TiO_2_ modified with 1 and 6 g/g of MPTES/TiO_2_.

Figure 1 compares the FTIR spectra of unmodified TiO_2_, MPTES, and TiO_2_ modified with 1 and 6 g/g MPTES. Upon modification with MPTES, a key observation is the notable reduction in the O–H bending (1600–1700 cm^–1^) and O–H stretching (3000–3700 cm^–1^) vibrations of TiO_2_ nanoparticles. This decrease indicates that surface hydroxyl groups on TiO_2_ reacted with MPTES during functionalization. Even at the lower ratio of 1 g/g, a significant reduction in the O–H signal is observed, and this effect is further amplified at the higher 6 g/g ratio, suggesting increased MPTES coverage on the TiO_2_ surface. Therefore, using higher MPTES concentration, higher functionalization density was obtained.

To illustrate the additional spectral changes introduced by MPTES functionalization, Figure 2 compares the zoom areas of FTIR spectra of unmodified TiO_2,_ neat MPTES, and TiO_2_ modified with 6 g/g MPTES. Several distinct vibrational bands characteristic of MPTES are observed in the modified TiO_2_ spectrum, supporting successful surface grafting. In particular, the emergence of bands in the 1000–1040 cm^–1^ region (Figure 2a) corresponds to Si–O–Si and Si–O–Ti stretching vibrations. These bands arise from condensation reactions between the hydrolyzed silanol groups of MPTES and either neighboring silanol groups (forming Si–O–Si bridges) or surface hydroxyl groups on TiO_2_ (forming covalent Si–O–Ti linkages), confirming chemical attachment to the nanoparticle surface. Additional spectral features include C–H stretching bands in the 2850–2950 cm^–1^ range (Figure 2c), while the S–H stretching vibration observed near 2570 cm^–1^ (Figure 2b) confirms the presence of the organic mercaptopropyl functionality introduced by MPTES. Although the intensity of these bands in the modified TiO_2_ spectrum is relatively low, likely due to the limited organic content and surface sensitivity of the technique, the detection of these bands, undouble demonstrate successful surface modification.

**Figure 2:**
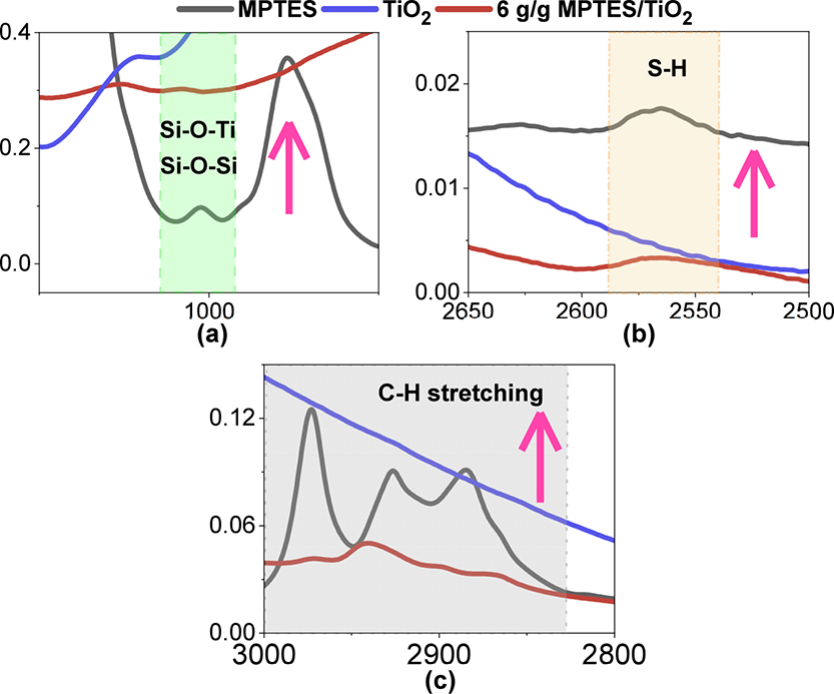
Zoomed areas from FTIR spectra of unmodified TiO_2_, MPTES and TiO_2_ modified with 6 g/g of MPTES/TiO_2_.

Further confirmation of the successful functionalization of TiO_2_ with MPTES was provided by contact angle measurements. It is well-established that silyl modification enhances the hydrophobicity of TiO_2_ surfaces, often leading to significantly increased water contact angle.^31^ Unmodified TiO_2_, being highly hydrophilic, exhibited a negligible contact angle (Figure S3a), as the surface was wet completely. Upon modification with 1 g/g MPTES, the contact angle increased to 51° ± 9 (Figure S3b), and a further increase to 69° ± 7 was observed for the 6 g/g MPTES/TiO_2_ (Figure S3c). Taken together, the findings confirm that the TiO_2_ nanoparticles modified with 6 g/g MPTES/TiO_2_ are more densely functionalized than those modified at the lower ratio.

The same findings were found by measuring of zeta potential at low pH (∼2) on nanoparticle dispersions, which dropped from +30 mV for unmodified TiO_2_ to +15 mV with 1 g/g MPTES, and further to −5 mV with 6 g/g MPTES (Figure S4). This shift reflects the effect of MPTES incorporation on the surface charge of TiO_2_, confirming effective surface modification and the effect of the increased quantity of the MPTES.

All the analyses presented, beside that the functionalization was achieved, it was denser in case of 6 g/g of MPTES used for modification.

### Chemisorption of MMA and BA Monomers from Aqueous Monomer Solution

To evaluate feasibility and clarify the chemisorption mechanism under simplified conditions, adsorption experiments were first performed in aqueous MMA (525 ppm) and BA (502 ppm) solutions using TiO_2_ nanoparticles functionalized with increasing MPTES loadings (0, 1, and 6 g/g TiO_2_). The influence of nanoparticle dosage (0.2–2 wt %) and contact time (1–5 h) was also assessed.

As shown in Figure S5, pristine TiO_2_ removed only ∼40% of MMA and slightly more BA, with negligible dependence on nanoparticle concentration or contact time, indicating mostly weak physisorption. In contrast, MPTES-functionalized TiO_2_ displayed markedly higher monomer uptake, confirming that thiol groups participate in reactive capturing of MMA and BA. At the highest functionalization level (6 g/g MPTES), removal efficiencies reached ∼80% for MMA and ∼90% for BA within 1 h. Notably, increasing TiO_2_ concentration provided only marginal improvement, whereas higher thiol functionalization density produced a pronounced effect, demonstrating that monomer capture is governed primarily by reactive surface functionality rather than total surface area.

It is clear that a part of the adsorbed monomer will be attached only physically to TiO_2_ surface. To distinguish between monomers that are chemically bonded and physically adsorbed might be challenging. To achieve this, a solvent extraction method using ethanol was applied to the monomers loaded TiO_2_ particles after their exposure to the aqueous monomer solution. Figure S6 illustrates the adsorbed and desorbed concentrations of MMA and BA from the aqueous monomer solution at different mixing times. Ethanol desorption tests (Figure S6) further substantiated the chemisorption pathway. The majority of adsorbed monomer remained on the particles after extraction, indicating the formation of covalently bound surface species instead of reversible physical adsorption. Quantitative mass balances (Table S7) revealed that approximately 90% of the sequestered MMA and BA were chemically anchored to the MPTES/TiO_2_ surface, with only ∼10% physically retained. Collectively, these results confirm that thiol oxidation on MPTES-modified TiO_2_ generates thiyl radicals that react with unsaturated monomers to form stable surface-bound adducts, thus, validating the mechanistic basis for subsequent application in more complex waterborne coatings.

### Chemisorption of MMA and BA Monomers from MMA/BA Latex by TiO_2_ Nanoparticles

MMA/BA latex was prepared using semi continuous emulsion polymerization, resulting in a stable aqueous dispersion with an average particle diameter of 137 nm, a total solids content of 49.3%, and a gravimetrically determined monomer conversion of 98.6%. GC analysis indicated residual monomer concentrations of 363 ppm for MMA and 1615 ppm for BA. In this system, the presence of polymer particles presents additional challenges for monomer removal, as residual monomers are primarily located within the polymer particles, while TiO_2_ nanoparticles will be placed in aqueous phase of the latex.

The influence of functionalization density (0, 1, and 6 g/g MPTES/TiO_2_), adsorbent concentration (0.2, 1, and 2 wt % TiO_2_), and mixing time (1, 3, and 5 h) on the adsorption affinity toward MMA and BA monomers was systematically investigated, and the results are summarized in Figure 3. When unmodified TiO_2_ was employed (Figure 3a,b), adsorption efficiencies were relatively modest, approximately 40% for MMA and 56% for BA, with little variation observed as the TiO_2_ concentration increased. In contrast, the use of functionalized TiO_2_ markedly enhanced monomer adsorption. Among the parameters studied, functionalization density proved to be the most influential: with densely functionalized TiO_2_ (Figure 3e,f), nearly complete removal of BA was achieved, while MMA adsorption reached 90% when 2 wt % TiO_2_ nanoparticles were used. The adsorbent concentration itself appeared to have only a minor effect on adsorption efficiency, likely due to the increased probability of nanoparticle interactions and aggregation. Such aggregation limits the effectiveness of the adsorbents by decreasing the available adsorption sites.

**Figure 3:**
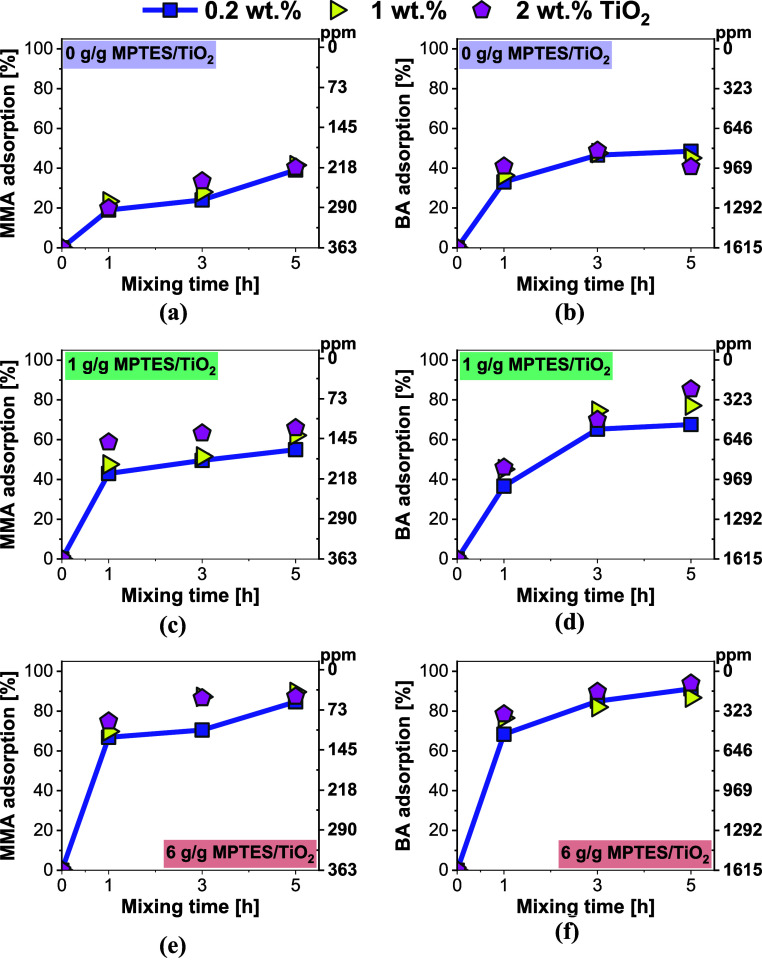
Influence of functionalization density, mixing time, and TiO_2_ concentration on the adsorption of MMA (a,c,e) and BA (b,d,f) in MMA/BA latex.

The difference between the adsorbed and desorbed concentrations of MMA and BA from the TiO_2_ in the latex is shown in Figure 4a,b, respectively. With unmodified TiO_2_, both concentrations remained nearly the same, demonstrating primarily physical adsorption of the monomers onto TiO_2_ nanoparticles. In contrast, the MPTES-modified TiO_2_ exhibited a significantly higher amount of monomer adsorption compared to desorption, indicating a strong and stable interactions residual monomers-TiO_2_ throughout the reactive thiol groups. Table S8 provides an estimate of chemical versus physical adsorption, indicating that approximately 80% of MMA and 90% of BA were chemically bonded, with the remaining 20% and 10% physically adsorbed, respectively.

**Figure 4:**
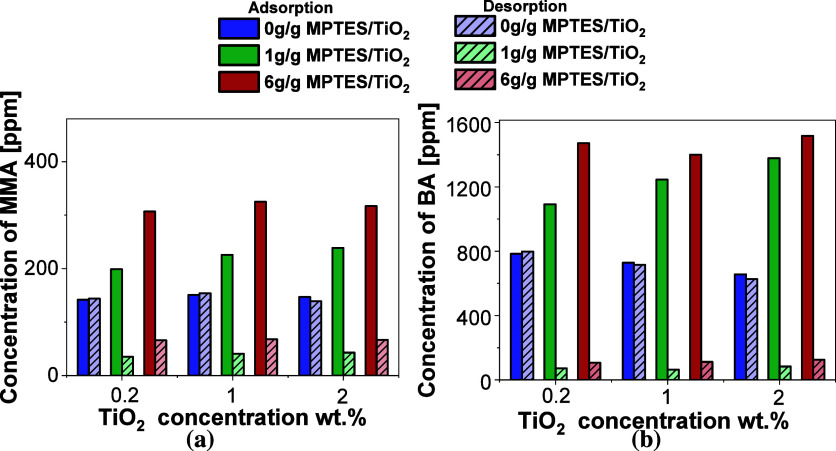
Adsorbed and desorbed concentration of (a) MMA and (b) BA for different modifier (MPTES) concentrations and different concentrations of TiO_2_ nanoparticles following 5 h of mixing in MMA/BA latex.

An unexpected yet significant finding of this study was the higher extent of chemisorption observed for BA compared to MMA, despite BA’s greater hydrophobicity and the initial assumption that adsorption would occur exclusively in the aqueous phase. Given that the TiO_2_ nanoparticles were dispersed in the latex, it was presumed that their interaction with monomers would be limited to the aqueous environment. However, the experimental results contradicted this expectation, suggesting a more complex adsorption mechanism involving interfacial TiO_2_ interactions with the polymer phase.

The results obtained so far suggest that the spatial localization of TiO_2_ nanoparticles within the polymer latex may play a critical role, as the interaction between TiO_2_ and residual hydrophobic monomers is likely influenced by their distribution. It is possible that unmodified TiO_2_, due to its hydrophilic character and limited colloidal stability, tends to remain aggregated in the aqueous phase, which would reduce the interfacial contact with the polymer particles. Under such conditions, diffusion of monomers from the polymer particles into the aqueous phase could become significant and thereby affect adsorption efficiency. This hypothesis is support by the observed colloidal stability of the TiO_2_ aqueous dispersions and the latex blends with TiO_2_ nanoparticles. Bimodal particle size distributions for both modified and unmodified TiO_2_ dispersions were observed by DLS (Figure S7). However, MPTES modification appeared to reduce nanoparticle aggregation due to decrease of the surface energy of the nanoparticles by addition of siloxane compound on its surface. As shown in Figure S7, although the bimodal particle size distribution was maintained in the modified particle dispersions, the distribution shifted toward smaller particle sizes, with the lower-size population decreasing from approximately 200 to 80 nm. This improved dispersion may be associated with enhanced electrostatic stabilization, as suggested by the more negative zeta potentials of −40 mV and −50 mV, respectively, measured for the modified dispersions at pH 11 (Figure S4).

On the other hand, colloidal issues were visually observed after blending of polymer and TiO_2_ nanoparticle dispersions. As shown in Figure S8a, a phase separation was observed in the blend of latex with unmodified TiO_2_, with visible sedimentation occurring within 24 h. In contrast, blends incorporating MPTES-modified TiO_2_ demonstrated markedly improved stability, with less visible aggregation or settling over the same period. This effect was especially pronounced at higher MPTES concentrations (6 g/g, Figure S8c), where the nanoparticles remained uniformly dispersed and no precipitation was observed.

To explore more in detail the potential distribution of TiO_2_ nanoparticles within the latex, TEM imaging was performed on a reference latex and on a latex blend containing 2 wt % surface-modified TiO_2_ nanoparticles functionalized with 0, 1, and 6 g/g MPTES. In the neat latex (Figure 5a,b), polymer particles with diameters ranging from 80 to 250 nm were observed. Upon the addition of unmodified TiO_2_ (0 g/g MPTES, Figure 5c,d), large black aggregates became apparent (up to 0.6 μm), likely resulting from nanoparticle clustering driven by insufficient electrostatic stabilization.

**Figure 5:**
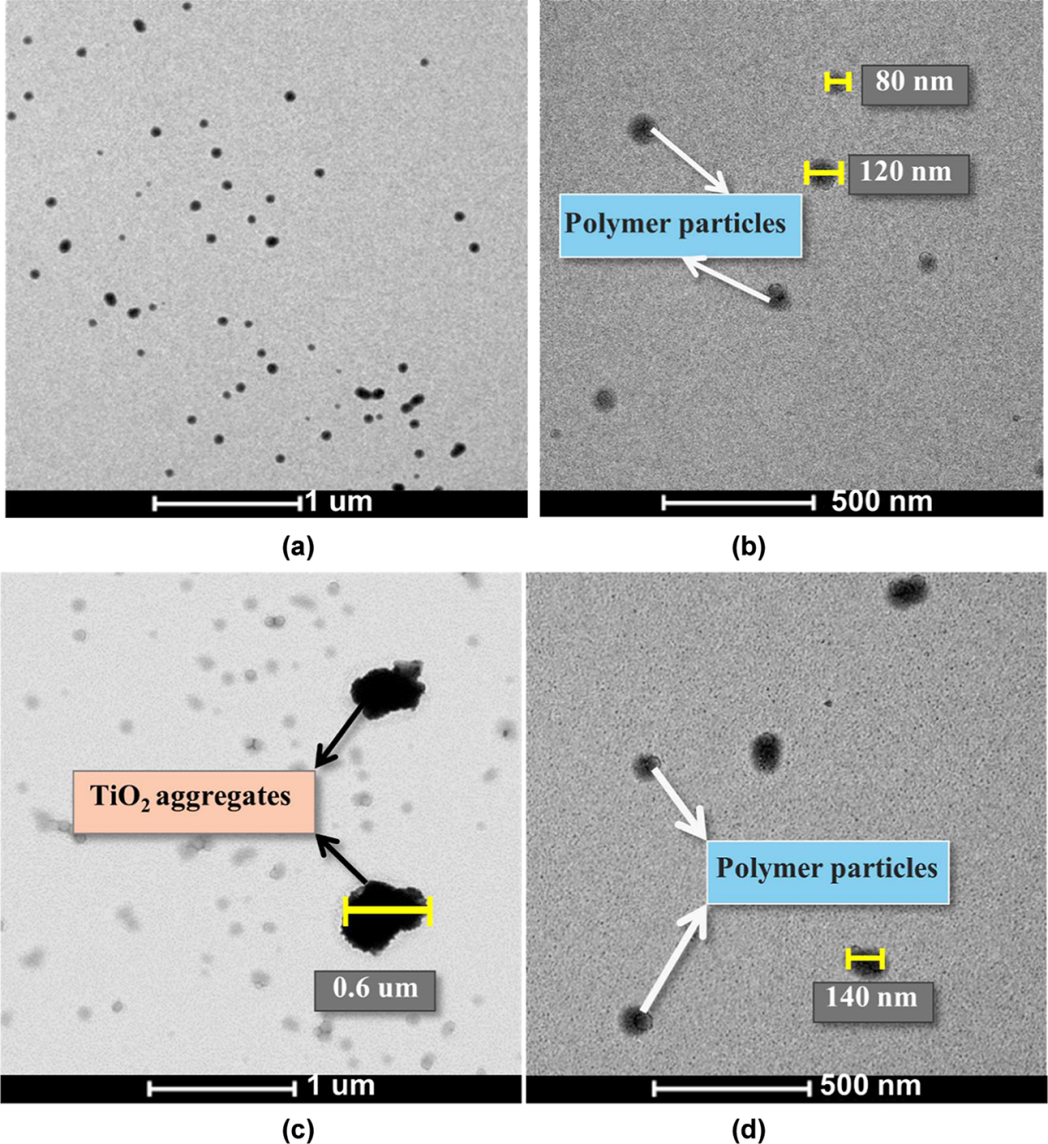
TEM images of (a,b) neat latex particles and (c,d) particles from latex incorporating 2 wt % unmodified TiO_2_ nanoparticles.

These improvements in colloidal stability were clearly observed in the TEM micrographs of latexes containing MPTES-modified TiO_2_. In these samples (Figure 6a,c), the absence of nanoparticle aggregation confirmed that surface modification promoted a uniform dispersion of TiO_2_ within the latex matrix. Higher-magnification images (Figure 7a,b) revealed that the overall morphology of the neat latex and the latex containing MPTES-modified TiO_2_ was similar. The darker, cloudy region surrounding the particles corresponds to the surfactant layer. However, a noticeably thicker black TiO_2_-rich shell was observed in the case of the TiO_2_-containing latex, indicating the presence of nanoparticles located at the particle surface. This interfacial arrangement is attributed to the amphiphilic nature of the modified TiO_2_. The MPTES molecule contains a triethoxysilane group that forms covalent siloxane (Si–O–Ti) bonds with the TiO_2_ surface, anchoring the organic modifier to the inorganic core. The terminal thiol-functionalized hydrocarbon chain extends outward, enhancing compatibility with the surrounding organic polymer. This structural duality imparts both hydrophilic (inorganic) and hydrophobic (organic) character to the nanoparticle surface, enabling the modified TiO_2_ to preferentially position at the polymer–water interface. As a result, the nanoparticles become anchored at the latex particle surface rather than remaining dispersed in the aqueous phase, leading to stable interfacial localization and improved colloidal stability. This interfacial positioning is critical, as it enables direct contact between the surface thiol groups and residual hydrophobic monomers (Scheme 1). BA, being more hydrophobic than MMA, is more highly concentrated in the polymer particles, explaining the significantly higher chemisorption observed for BA (Figure 3).

**Figure 6:**
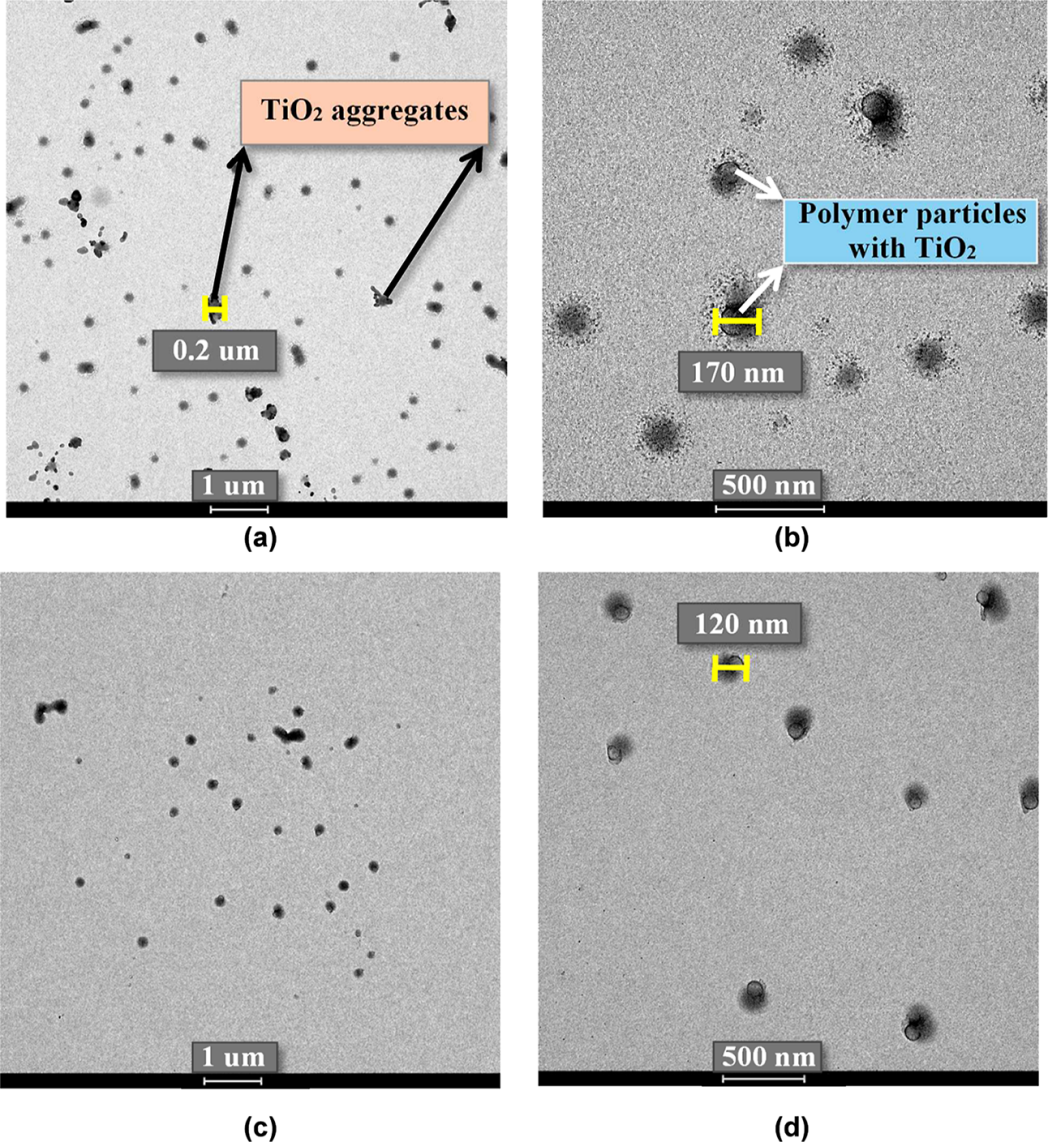
TEM images of latex particles incorporating 2 wt % of TiO_2_ nanoparticles modified with (a,b) 1g/g MPTES/TiO_2_ and (c,d) 6g/g MPTES/TiO_2_.

**Figure 7:**
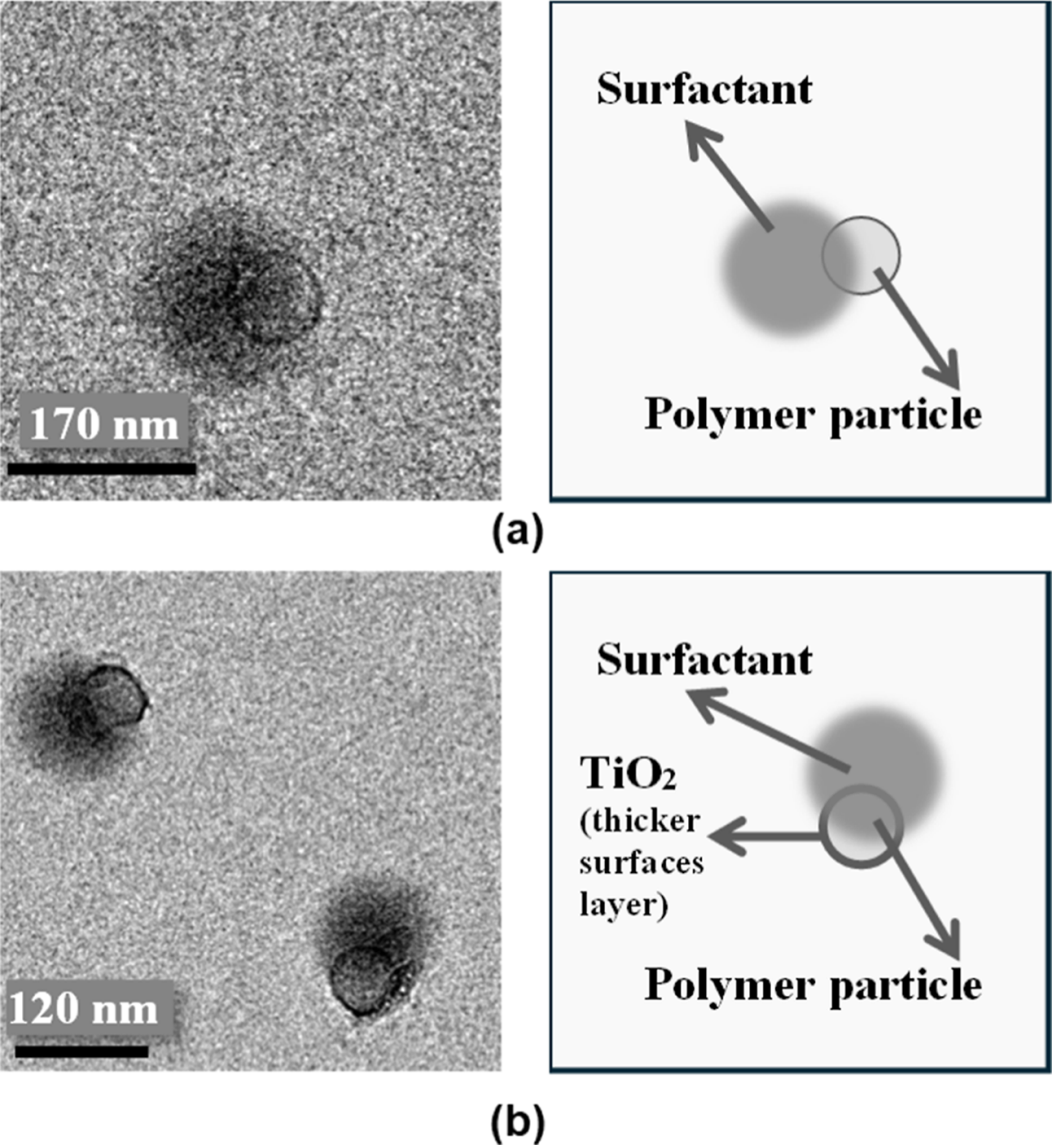
Zoomed TEM images and corresponding scheme of (a) neat MMA/BA particle; and (b) hybrid particle from dispersion containing 2 wt % TiO_2_ nanoparticles modified with 6 g/g MPTES/TiO_2_.

**Scheme 1:**
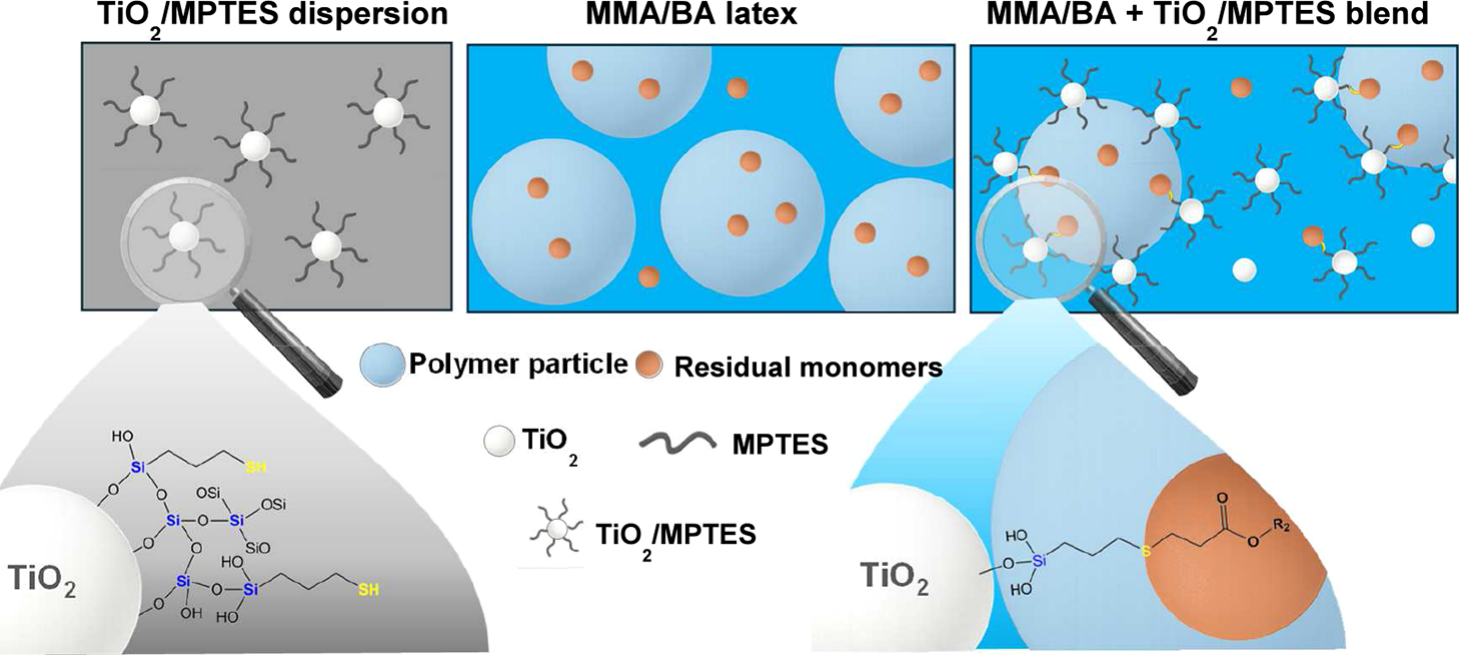
Schematic Illustration of the Chemisorption Mechanism Occurring in MMA/BA Latex upon Addition of MPTES Modified TiO_2_ Nanoparticles

These results highlight that surface modification not only enhances nanoparticle dispersion but also drives selective interfacial positioning, which is essential for maximizing monomer scavenging efficiency

### Film Characteristics and Performance

Polymer and composite films were prepared from the neat latex and blends containing 2 wt % of either unmodified or modified TiO_2_ by water evaporation at controlled atmospheric conditions (23 °C and 55% active humidity). The morphology of the films’ cross-section was analyzed by SEM imaging and EDX mapping. As shown in Figure 8a,b,d,f, the TiO_2_ particles are not clearly visible in the SEM micrographs due to their nanoscale dimensions. However, EDX mapping confirmed the presence and distribution of titanium within the films (green dots), as illustrated in Figure 8c,e,g.

**Figure 8:**
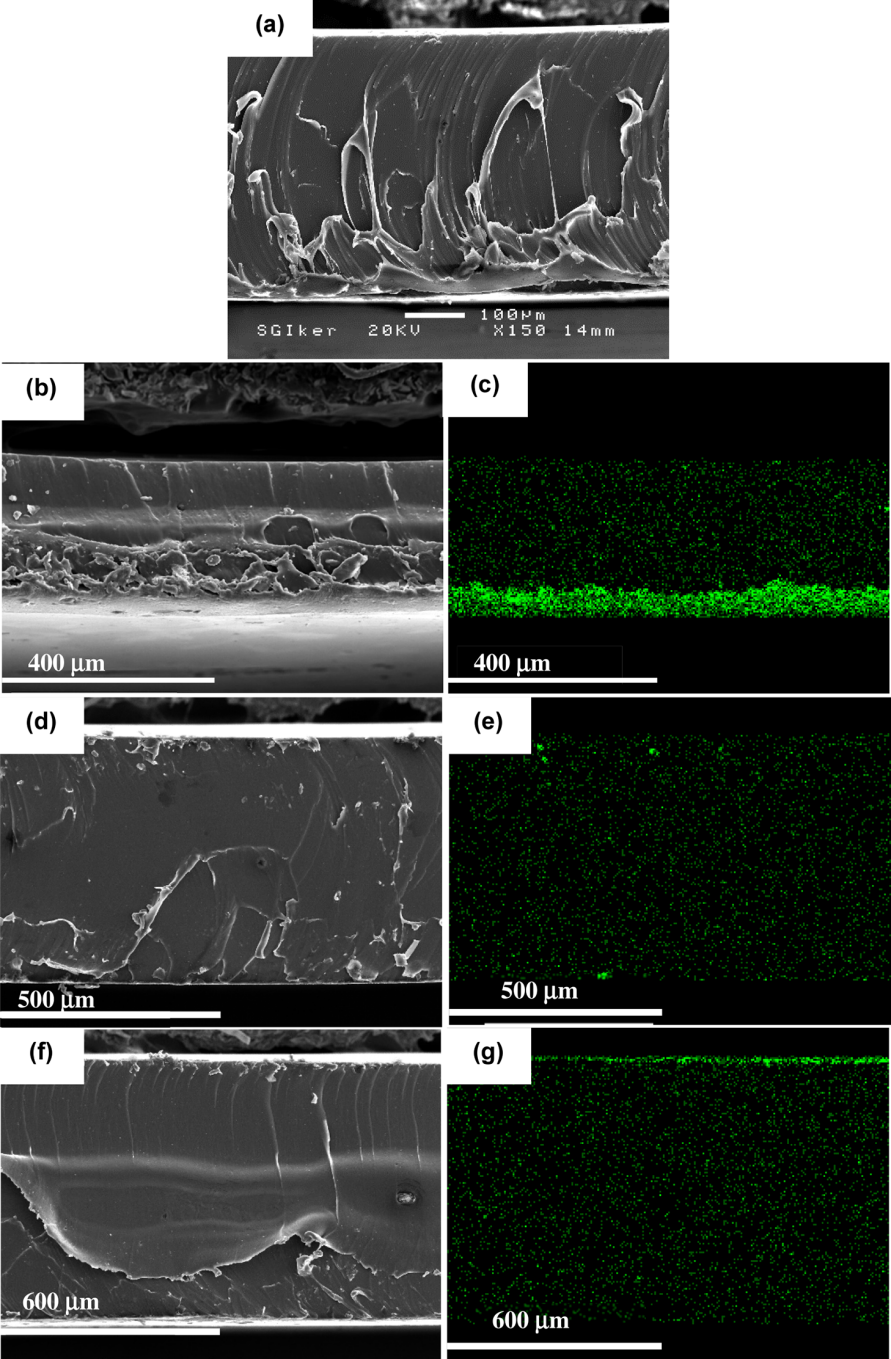
SEM images and corresponding Ti EDX map of film cross sections of: (a) MMA/BA polymer film; (b,c) film containing 2 wt % unmodified TiO_2_; (d,e) film containing 2 wt % modified TiO_2_ with 1 g/g MPTES/TiO_2_; and (f,g) film containing 2 wt % modified TiO_2_ with 6 g/g MPTES/TiO_2_.

In the film containing unmodified TiO_2_, the nanoparticles appeared to precipitate and accumulate near the bottom of the film (Figure 8c), as evidenced by the strong titanium signal. In contrast, the dispersion and distribution of TiO_2_ improved significantly when modified nanoparticles were used. Although the latex containing the less densely modified TiO_2_ (1 g/g MPTES/TiO_2_) still exhibited some aggregates within the polymer matrix (Figure 8e), the more densely functionalized nanoparticles (6 g/g MPTES/TiO_2_) displayed a much more uniform distribution (Figure 8g). However, a thin TiO_2_-rich layer was observed at the film surface in Figure 8g, indicating that certain self-stratification of TiO_2_ nanoparticles occurred during film formation. The micrometer-thick surface layer (Figure 8g) appeared monophasic, suggesting that individual TiO_2_ nanoparticles were stratified. The self-stratification phenomenon has been reported to occur in bimodal latex blends when there is a sufficient size difference between large and small particles under specific conditions.^33,34^ In the present case, stratification takes place between the large polymer particles and the much smaller, densely functionalized TiO_2_ nanoparticles. We hypothesize that, under conditions of high surfactant concentration, some surfactant molecules may desorb from the polymer particle surfaces—particularly after adsorption of the densely modified TiO_2_ nanoparticles, which themselves provide additional colloidal stabilization. This excess surfactant likely stabilizes a portion of the TiO_2_ nanoparticles, preventing their aggregation. During slow water evaporation, when diffusion of the larger polymer particles dominates over the evaporation rate, the smaller TiO_2_ nanoparticles accumulate at the film surface.^32^ In contrast, for the 1 g/g MPTES/TiO_2_ modification, the nanoparticles are less colloidally stable and provide weaker stabilization to the polymer particles. As a result, they tend to aggregate rather than undergo stratification.

The performance of the MMA/BA polymer film for coating and paint applications was evaluated by measuring water sensitivity and mechanical resistance, two key properties directly related to the durability and overall resistance of the coating film. Composite films containing 2 wt % TiO_2_ nanoparticles were compared with the neat polymer film.

Water sensitivity was evaluated by monitoring the water uptake of the films during prolonged immersion. As shown in Figure 9a, the film containing unmodified TiO_2_ exhibited more than 20% higher water uptake compared to the neat polymer film, increasing from approximately 50% to 75% after 18 days of immersion. This increase can likely be attributed to the hydrophilic nature of unmodified TiO_2_ and its aggregates, which may act as reservoirs that elevate the osmotic pressure and thereby drive greater water absorption within the film matrix. In contrast, surface modification of TiO_2_ reduced this increase in water sensitivity. Notably, when TiO_2_ nanoparticles were more densely functionalized, the water uptake approached that of the neat polymer film (less than 5% increase, from 50% to 54%). This behavior is likely due to the decreased hydrophilicity of TiO_2_ nanoparticles modified with 6 g/g MPTES and the absence of hydrophilic aggregates, as observed in the TEM images (Figure 6).

**Figure 9:**
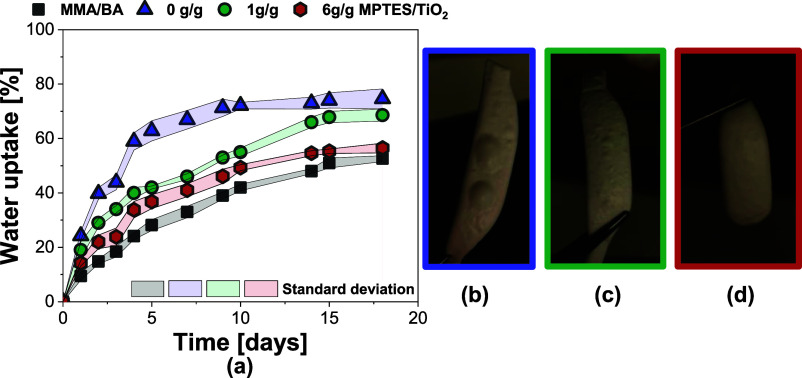
(a)­Water uptake of polymer films containing 2 wt % TiO_2_ nanoparticles, comparing modified and unmodified TiO_2_; photos after water immersion of the films containing 2 wt % TiO_2_, modified with MPTES/TiO_2_ ratios of: (b) 0 g/g, (c) 1 g/g, and (d) 6 g/g.

Another noteworthy observation was the formation of hydrated macrodomains, or water pockets, within the films following immersion in water (Figure 9b–d). These features were particularly pronounced in samples containing unmodified TiO_2_ (Figure 9b). In contrast, films incorporating modified TiO_2_ showed significantly less or no presence of water pockets, particularly at the highest level of MPTES modification (6 g/g, Figure 9c).

The mechanical properties of the films containing 2 wt % of either unmodified TiO_2_ or modified TiO_2_ were evaluated by tensile test, and the results are shown in Figure 10 and in Table S9. The incorporation of TiO_2_ into the polymer acted as a reinforcing agent, leading to notable improvement of Young’s modulus (75%), offset yield stress (60%), and tensile strength (25%), particularly when the TiO_2_ was modified. This enhancement was higher with more densely functionalized TiO_2_ nanoparticles. The enhancement of mechanical resistance of polymer films containing modified TiO_2_ particles is synergistic effect of TiO_2_ and likely thicker siloxane shell around the particle, but also due to more uniform distribution of the modified TiO_2_ within the polymer matrix and reduced aggregation compared to unmodified particles. Although elongation at break decreased slightly from 450 ± 18 to 375 ± 20, this trade-off is typical when stiffness increases, resulting in films that are less flexible, yet stiffer overall.

**Figure 10:**
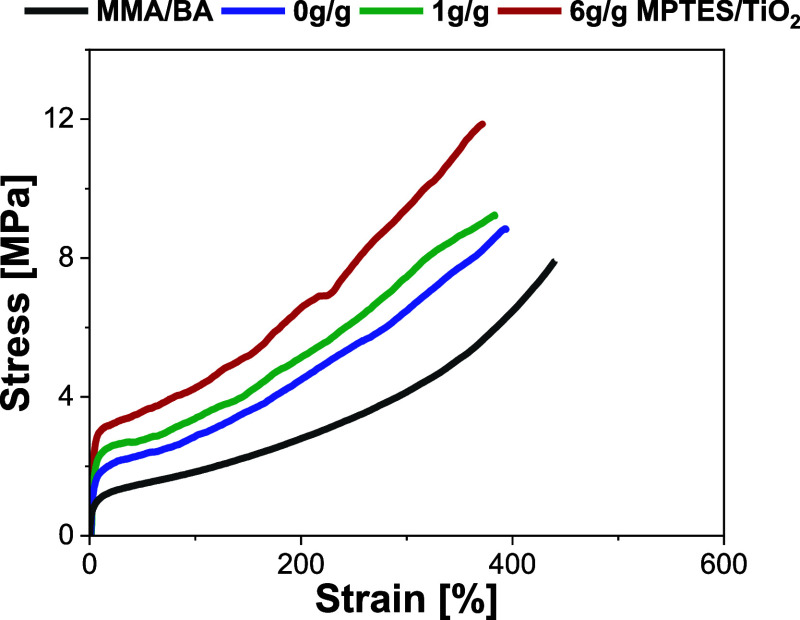
Mechanical properties of MMA/BA polymer film and blends containing 2 wt % TiO_2_ nanoparticles: comparison between unmodified and modified TiO_2_.

Additionally, the wettability of the films containing 2 wt % TiO_2_ nanoparticles, both modified and unmodified, was evaluated through contact angle measurements and compared to the neat polymer film. As shown in Figure S9, the incorporation of unmodified TiO_2_ led to a decrease in contact angle from 94° to 84°, consistent with the hydrophilic nature of neat TiO_2_. In contrast, surface modification with MPTES resulted in a significant increase in hydrophobicity. When 1 g/g MPTES/TiO_2_ was used, the contact angle recovered to values comparable to the neat polymer, while a higher modification degree (6 g/g MPTES/TiO_2_) further elevated the contact angle to 98°. This behavior reflects the enhanced hydrophobic character imparted by MPTES grafting, which is supported by the surface properties shown in Figure S3.

## Conclusions

This work establishes thiol-functionalized TiO_2_ nanoparticles as a multifunctional additive for controlling residual monomer content in MMA/BA polymer latexes. Surface modification with 3-(mercaptopropyl)­triethoxysilane (MPTES) introduced reactive −SH groups capable of radical generation in aqueous media, enabling covalent reaction with the C=C double bonds of methyl methacrylate (MMA) and butyl acrylate (BA). By coupling adsorption with chemical bonding, this strategy ensured stable immobilization of captured monomers on the nanoparticle surface.

Systematic evaluation of functionalization density, nanoparticle concentration, and mixing time revealed that dense MPTES coverage produced the highest reactivity. Under optimized conditions, modified TiO_2_ achieved up to 90% removal of MMA and near-complete removal of BA, lowering the overall residual monomer concentration from ∼1900 ppm to 120 ppm. Determining the desorbed quantity of monomers with ethanol from the monomer loaded TiO_2_ nanoparticles, indicated that 80–90% of the captured monomers were covalently bound, greatly reducing the potential for re-emission during film formation.

Colloidal and morphological characterization clarified the structural origins of this performance. DLS and zeta potential measurements showed that MPTES modification enhanced electrostatic stabilization and reduced aggregation, while TEM imaging revealed preferential localization of the nanoparticles at the polymer particle surfaces, where residual monomers are concentrated. This interfacial positioning maximizes contact between reactive sites on the TiO_2_ and the monomer-rich polymer phase, thereby promoting efficient capture.

Importantly, incorporation of the modified nanoparticles did not compromise water resistance of the resulting films, while mechanical testing demonstrated a 75% increase in Young’s modulus relative to neat polymer films. This simultaneous improvement in environmental compatibility and mechanical integrity highlights the synergy between chemical functionality, particle dispersion, and interfacial interactions.

Overall, thiol-functionalized TiO_2_ provides a materials-based solution for lowering volatile organic compound emissions from waterborne polymer latexes while reinforcing film properties. The ability to integrate pigmentary TiO_2_ functionality with reactive nano adsorption opens a pathway toward next-generation low-VOC pigmented coatings that combine optical performance, chemical reactivity, and mechanical durability.

## Supplementary Material



## Notes

Formulation of MMA/BA latex; modification reactions of TiO_2_ with MPTES; preparation of TiO_2_ dispersions blended with aqueous monomer solution and MMA/BA latex; calibration curves for MMA and BA residual monomers; GC chromatogram; table of FTIR characteristic bands; images of contact angle measurements of modified and unmodified TiO_2_; zeta potential plot; percentage of physical and chemisorption by TiO_2_ in aqueous monomer solution and in MMA/BA latex; particle size distribution of TiO_2_ dispersions; images illustrating stability of TiO_2_/latex blends; tensile test and contact angle results of TiO_2_/polymer films (PDF)
