# Supplementary material for: Thiol-Functionalized TiO2 as Reactive Nanoadsorbents for Residual Monomer Removal from Waterborne Polymer Dispersions
Source: Ind Eng Chem Res. 2026 Jan 19;65(3):1483–95. doi: 10.1021/acs.iecr.5c04301 (PMC12856996; doi:10.1021/acs.iecr.5c04301)
Supplement: Supplementary file 1 [file ie5c04301_si_001.pdf]

Supporting Information

Thiol-Functionalized TiO_2 as Reactive Nanoadsorbents for Residual Monomer Removal from Waterborne Polymer Dispersions

Ana Trajcheva[†], Pablo Morales[†], Justine Elgoyhen[†], and Radmila Tomovska[†,§]*

[†]POLYMAT and Departamento de Química Aplicada, Facultad de Ciencias Químicas, University of the Basque Country UPV/EHU, Joxe Mari Korta Zentroa, Tolosa Etorbidea 72, Donostia-San Sebastián 20018, Spain

[§]IKERBASQUE, Basque Foundation for Science, Plaza Euskadi 5, 48009, Bilbao, Spain

*Corresponding author e-mail address: radmila.tomovska@ehu.eus

Synthesis of MMA/BA latex

Table S1. Formulation of 50% p(MMA/BA) latex obtained via semicontinuous seeded emulsion polymerization.

Component	Initial charge [g]	Feed [g]	Initiating phase [g]
**Batch polymerization of MMA/BA seed with 20% s.c**			
MMA	65.4	-	-
BA	66	-	-
Water	516.3	-	-
Dowfax 21A (3 wt. % bm)	8.4	-	-
KPS (0.5 wt. % bm)	0.6	-	-
**Semicontinuous polymerization of MMA/BA latex with 50% s.c**			
Seed	656.7	-	-
MMA	-	402	-
BA	-	407	-
Dowfax 21A (3 wt. % bm)	-	52.6	-

KPS (0.5 wt. % bm)	-	-	4.3
Water	-	254	163

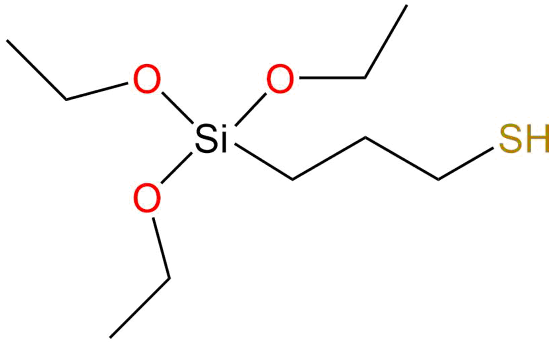

Scheme S1. Chemical structure of MPTES.

Table S2. Formulation of modification reactions of TiO_2 with MPTES.

MPTES/TiO_2 g/g	TiO_2 [g]	MPTES [g]	Butanone [g]	Yield of modified TiO_2 [%]
1	6.0265	6.3383	50.4106	86.3
6	6.0449	36.0520	50.1587	84.6

Table S3. Composition of 20 wt.% aqueous dispersions containing unmodified TiO_2 and TiO_2 modified with MPTES.

MPTES/TiO_2 g/g	TiO_2 [g]	Mili Q water [g]
0	4.1448	20.2555
1	4.0265	20.3383
6	4.0449	20.0520

Table S4. Formulation of blends of TiO_2 dispersion and aqueous monomer solutions for adsorption of dissolved monomers

MPTES/TiO_2 g/g	wt.% of TiO_2	20 wt.% TiO_2 dispersion[g]	Aqueous monomer solution [g]
0	0.2	0.2369	20.1869
	1	1.0698	20.2236
	2	2.0397	20.1169
1	0.2	0.2136	20.2369
	1	1.1065	20.4369
	2	2.2639	20.1023

6	0.2	0.2265	20.0369
	1	1.2062	20.5639
	2	2.1564	20.1256

Table S5. Formulation of TiO_2 dispersions and MMA/BA polymer latex for adsorption of residual monomers

**MPTES/TiO_2 g/g**	**wt.% of TiO_2**	**20 wt.% TiO_2 dispersion[g]**	**MMA/BA latex [g]**
0	0.2	0.2104	20.6086
	1	1.1384	20.1255
	2	2.2564	20.0362
1	0.2	0.2205	20.5011
	1	1.0853	20.2553
	2	2.3042	20.5083
6	0.2	0.2358	20.1623
	1	1.2132	20.6856
	2	2.2668	20.3585

Calibration curves

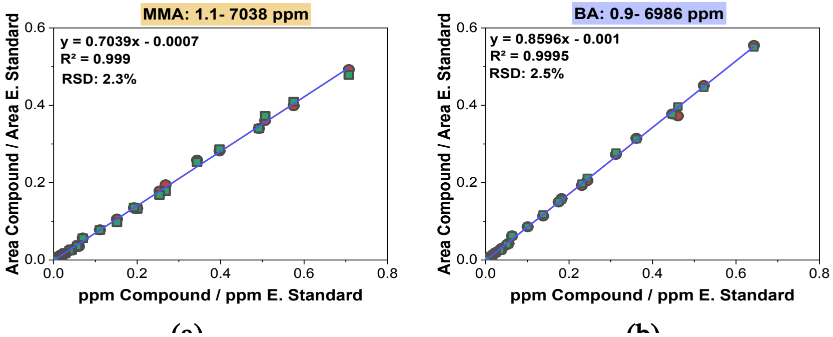

Figure S1. GC calibration curves of: a) MMA and b) BA.

Evaporation of MMA and BA from TiO_2 during drying

To evaluate the potential evaporation of the monomers previously adsorbed onto non-modified TiO_2, an experiment was conducted using an aqueous monomer solution containing MMA, and BA. TiO_2 nanoparticles were introduced into the solution and stirred for 5 hours to allow for monomers adsorption. Following this, the monomer-loaded TiO_2 was separated by centrifugation

at 5000 rpm for 30 minutes. Despite the separation, a small volume of the aqueous monomer solution—approximately 1.6 mL—remained with the TiO_2 particles, as complete removal would require heating, which could simultaneously trigger the evaporation of the adsorbed monomers.

To investigate whether monomers adsorbed onto TiO_2 would desorb and evaporate over time, two sealed vials were prepared: one containing the separated TiO_2 nanoparticles with the remaining 1.6 mL of aqueous monomers solution, and the other containing only 1.6 mL of the same monomer solution (

Figure S2). Both vials were stored at 25%˚C for four days. After this period, the vapor phase in the headspace of each vial was qualitatively analyzed using GC. As shown in

Figure S2, the chromatographic profile of the vial containing the TiO_2 revealed a clear presence of MMA and BA in the vapor phase (red curve), indicating that monomers initially adsorbed onto the TiO_2 were released over time.

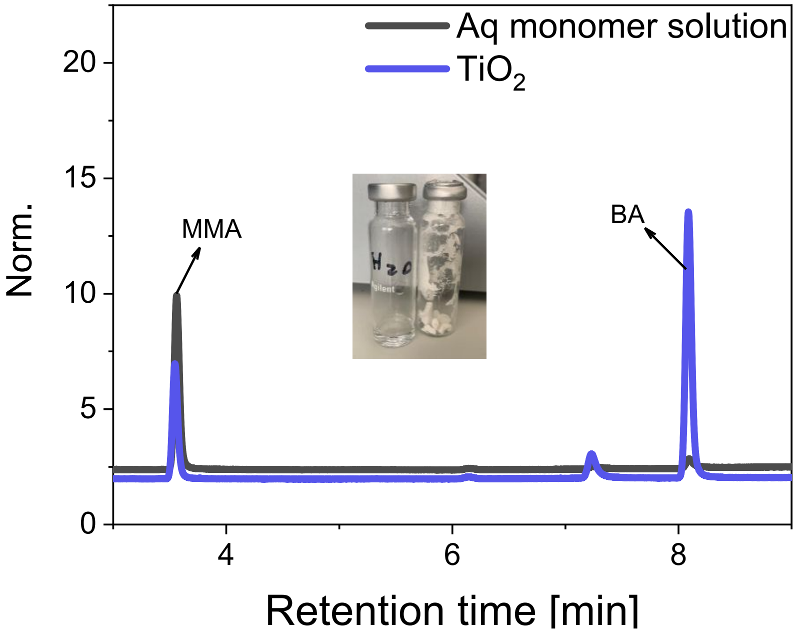

Figure S2. GC analysis of vapor phase extracted from a vial containing 1.6 mL of MMA and BA saturated aqueous solution (black line); and 1.6 mL of MMA and BA saturated aqueous solution in which TiO_2 loaded with adsorbed monomers was added (blue line).

FTIR characteristic bands of MPTES and TiO_2

Table S 6. Characteristic FTIR bands of MPTES and TiO_2.

Wavelength [cm^{-1}]	MPTES	TiO_2	MPTES modified TiO_2
3000-3700	-	O-H stretching	O-H stretching (decreased)
2550-2570	S-H stretching	-	S-H stretching
2850-2950	C-H stretching	-	C-H stretching
1600-1700	-	O-H bending	O-H bending (decreased)
1000-1040	Si-O-C	-	Si-O-Ti/Si-O-Si
~750	Si-C	-	Si-C
500-700	-	Ti-O	Ti-O

Contact angle results

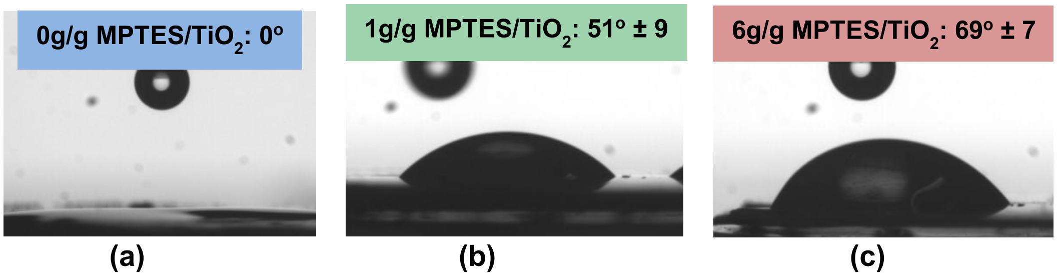

Figure S3. Contact angles of a) TiO_2, b) 1 g/g MPTES/TiO_2 and c) 6 g/g MPTES/TiO_2.

Zeta potential results

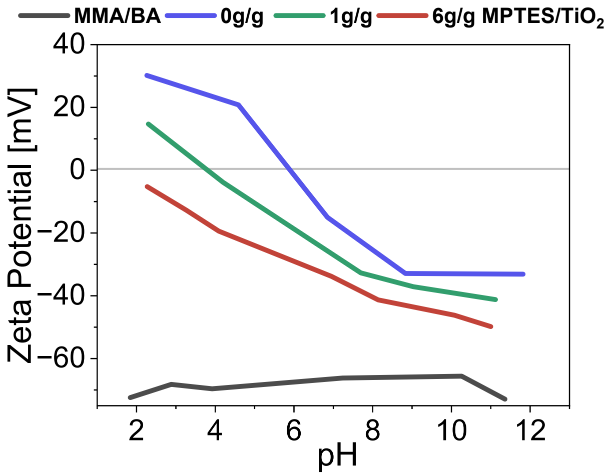

Figure S4. Zeta potential of MMA/BA latex, non-modified and modified TiO_2 with 1g/g and 6 g/g MPTES/TiO_2.

Efficiency of unmodified and MPTES-modified TiO_2 for adsorption of MMA and BA monomers in aqueous monomer solution

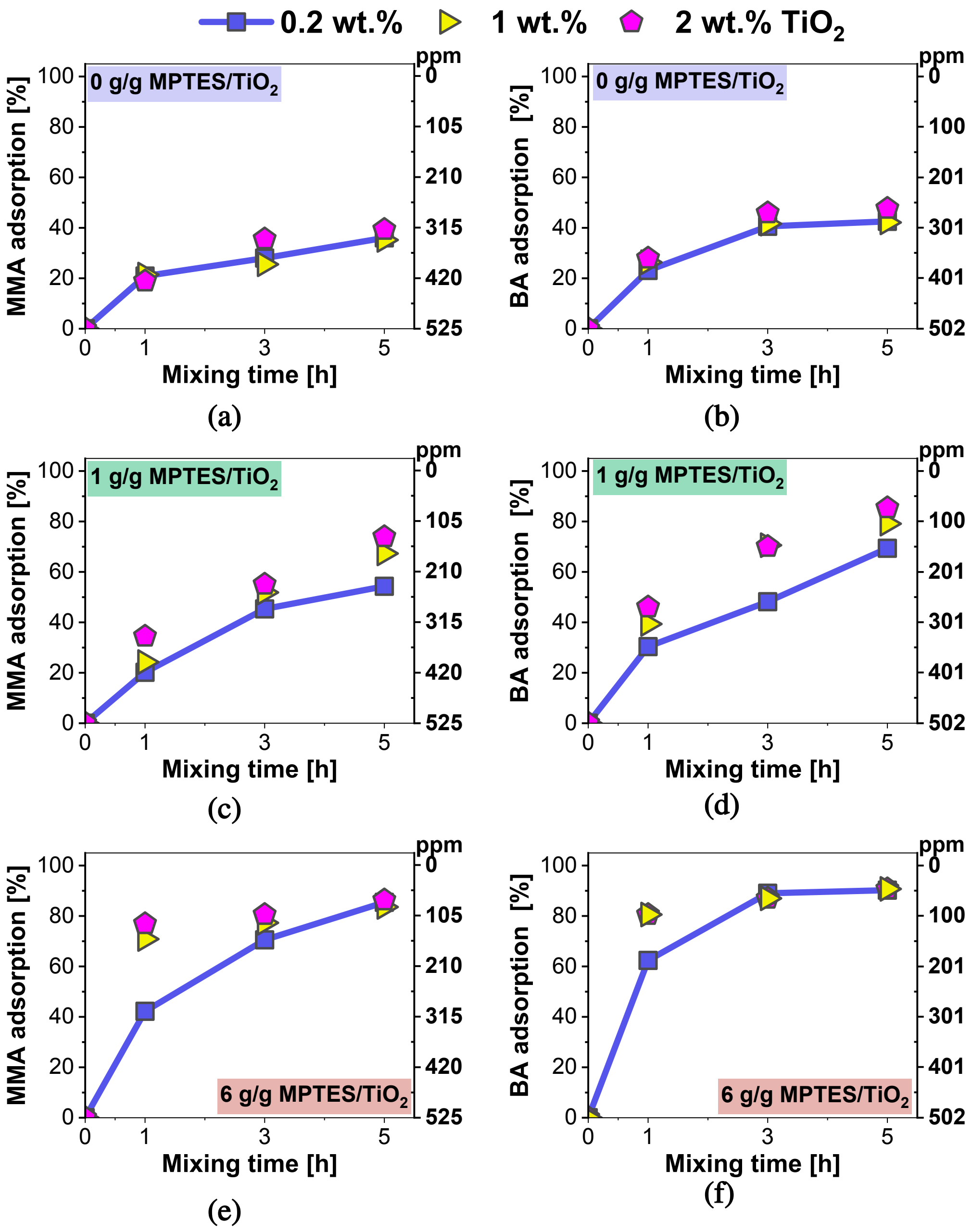

Figure S5. Influence of functionalization density, mixing time, and TiO_2 concentration on the adsorption of MMA (a, c and e) and BA(b, d and f) from aqueous monomer solution.

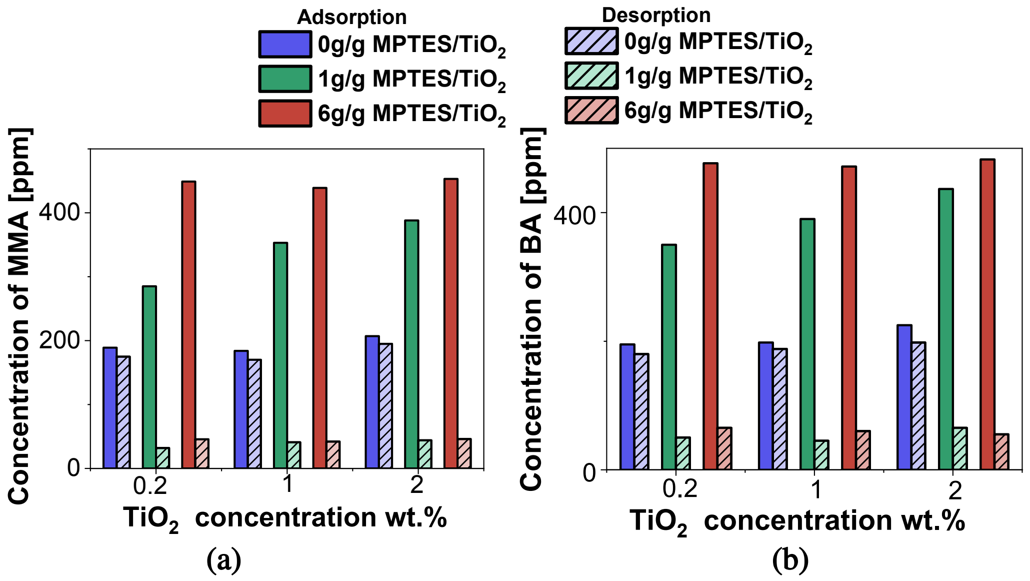

Figure S6. Adsorbed and desorbed concentration of a) MMA and b) BA after 5 h of mixing of monomers solution and TiO_2 with different functionalization density and various concentrations.

Table S7. Physical versus chemical adsorption by TiO_2 with different functionalization density and different concentrations after5h of aqueous monomer solution treatment.

TiO_2 wt.%	≈% of Adsorption of MMA				≈% of Adsorption of BA			
	1g/g MPTES		6g/g MPTES		1g/g MPTES		6g/g MPTES	
	Physical	Chemical	Physical	Chemical	Physical	Chemical	Physical	Chemical
0.2	11	89	10	90	12	88	13	87
1	12	88	10	90	12	88	12	88
2	11	89	10	90	13	87	11	89

Efficiency of bare and modified TiO_2 for adsorption of MMA and BA monomers in MMA/BA latex

DLS results of 0.05 wt.% TiO_2 water dispersions

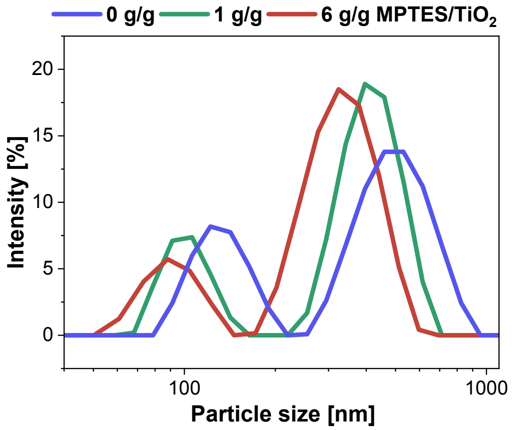

Figure S7. Particle size distribution of 0.05 wt.% TiO_2 water dispersions: TiO_2 modified with 0 g/g, 1 g/g and 6 g/g MPTES/TiO_2.

Table S8. Physical versus chemical adsorption by TiO_2 with different functionalization density and different concentrations after5h of MMA/BA latex treatment.

TiO_2 wt.%	≈% of Adsorption of MMA				≈% of Adsorption of BA			
	1g/g MPTES		6g/g MPTES		1g/g MPTES		6g/g MPTES	
	Physical	Chemical	Physical	Chemical	Physical	Chemical	Physical	Chemical
0.2	18	82	21	79	7	93	7	93
1	18	82	20	80	5	95	8	92
12	18	82	21	79	6	94	8	92

Stability of the polymer latex upon treatment with bare and modified TiO_2

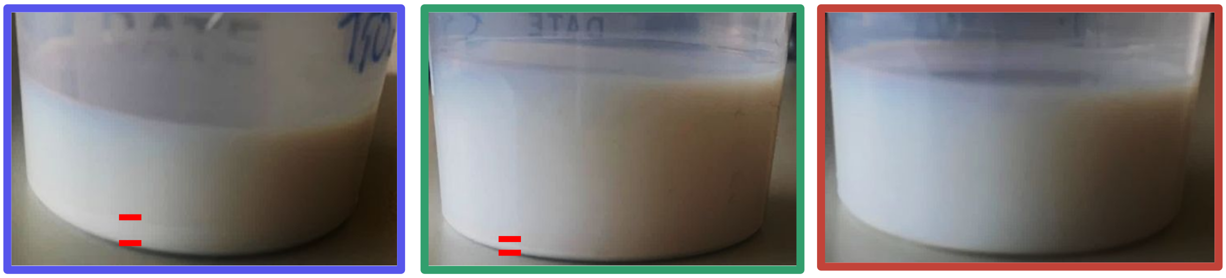

(a) (b) (c)

Figure S8. Appearance of TiO_2/latex blends after treatment (TiO_2 particles separation is marked by red lines): (a) bare TiO_2 (0 g/g MPTES/TiO_2), (b) 1 g/g MPTES/TiO_2, and (c) 6 g/g MPTES/ TiO_2.

Tensile test results of polymer and hybrid films

Table S9. Mechanical properties of MMA/BA polymer films without and with 2 wt.% unmodified and modified TiO_2 nanoparticles.

Sample	Young's Modulus [MPa]	Offset Yield Stress [MPa]	Tensile strength [MPa]	Elongation at break [%]
**MMA/BA**	0.16	1	8.7±0.2	450±18
**0 g/g MPTES/TiO_2**	0.2	1.4	8.8±0.3	395±32
**1 g/g MPTES/TiO_2**	0.48	1.8	9.3±0.2	385±28
**6 g/g MPTES/TiO_2**	0.65	2.5	12±0.4	375 ±20

Contact angle measurements for neat polymer and polymer blends containing modified and unmodified TiO_2 nanoparticles

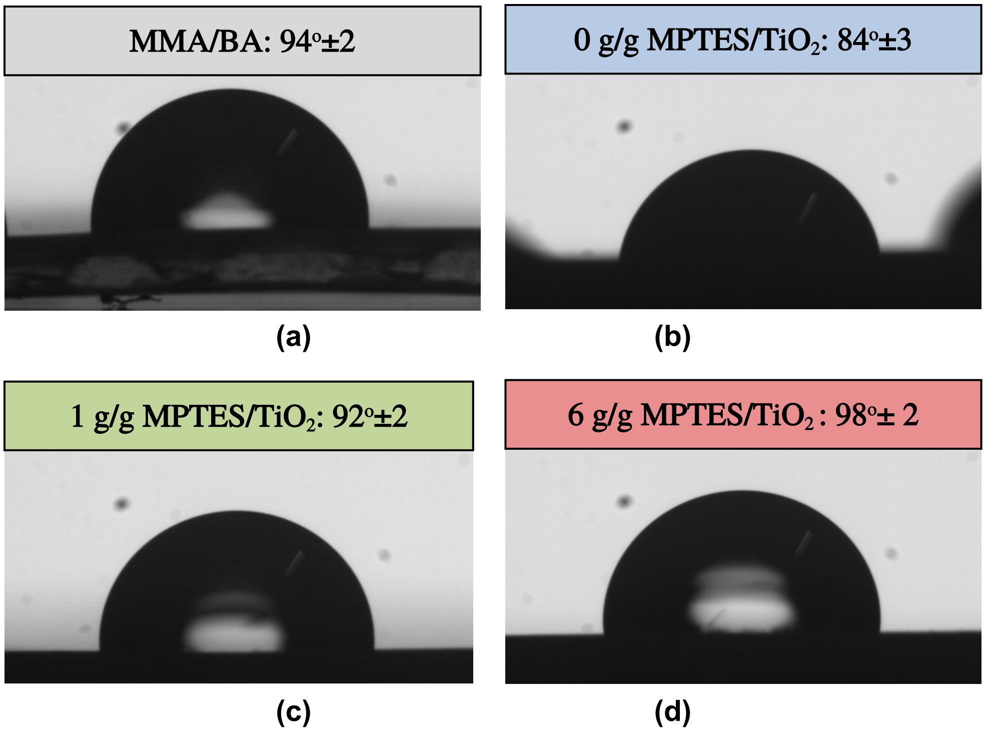

Figure S9. Contact angles of a) MMA/BA film, and MMA/BA films containing 2 wt.% of: b) 0 g/g, c)1 g/g and d) 6 g/g TiO_2/MPTES, recorded after water-washing to remove surfactant.
